# Synthesis of Novel Stable ZnSe Magic‐Sized Clusters and Their Direct Transformation to Quantum Dots

**DOI:** 10.1002/advs.75439

**Published:** 2026-05-04

**Authors:** Bin Song, Zihui Jin, Xuanyu Zhang, Rong‐Jun Xie

**Affiliations:** ^1^ Fujian Key Laboratory of Surface and Interface Engineering For High Performance Materials College of Materials Xiamen University Xiamen P. R. China; ^2^ College of Materials Science and Engineering Science and Education Integration College of Energy and Carbon Neutralization Zhejiang University of Technology Hangzhou P. R. China; ^3^ School of Physics and Optoelectronic Engineering Hangzhou Institute for Advanced Study University of Chinese Academy of Sciences Hangzhou P. R. China

**Keywords:** low‐temperature synthesis, magic‐size clusters, quantum dots, stable, ZnSe

## Abstract

The inherent instability of magic‐size clusters (MSCs), stemming from their ultrasmall dimensions, presents a significant challenge for their synthesis and practical application. This study developed a low‐temperature synthesis strategy employing a coordination system based on 1‐octadecene selenium (ODESe), diphenylphosphine (DPP) and zinc carboxylate precursors, which successfully produced novel ZnSe clusters with excellent thermal and ligand stability. These robust clusters can serve as precursors for the direct transformation into ZnSe quantum dots (QDs) under mild conditions by simply adding a Se precursors. Notably, the resulting cluster‐transformed QDs (6.3 nm) are significantly larger in size than those obtained through the conventional hot‐injection method (4.3 nm) at the identical reaction temperature, corresponding to a 19 nm redshift in emission. This work not only establishes a new paradigm for synthesis of stable MSCs but also reveals a non‐classical growth regime from robust clusters to large‐sized QDs, offering profound insights into the nucleation and growth mechanisms of nanocrystals.

## Graphical abstract

We have developed a low‐temperature synthetic approach that enables the fabrication of novel ZnSe MSCs with remarkable thermal stability. These stabilized MSCs serve as efficient precursors that can be readily converted to ZnSe QDs under mild conditions through simple introduction of a selenium source.

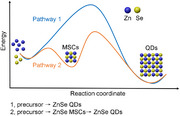

## 1 Introduction

Colloidal semiconductor QDs have been extensively studied over the past four decades as a prominent class of functional nanomaterials, [1, 2, 3, 4, 5, 6, 7] finding widespread applications in displays [8], energy conversion [9] and biomedicine [10]. In contrast, magic‐sized clusters (MSCs), often emerging as byproducts during QD synthesis, have attracted comparatively limited research attention [11, 12, 13, 14, 15, 16, 17, 18]. Nevertheless, MSCs play a critical role in elucidating early stages of nanocrystal formation, serving as key intermediates in transformation from molecular precursors to QDs [19, 20, 21, 22, 23, 24, 25, 26, 27, 28]. Unlike conventional QDs, MSCs typically exhibit narrower absorption profiles, a consequence of their discrete sizes and well‐defined atomic structures [24, 29, 30, 31, 32]. This structural precision not only confers distinct optical properties but also enhances thermodynamic stability in certain MSC species. Owing to their unique structure and growth nature, MSCs have been strategically utilized as precursors or seeds for directing material transformations in nanocrystal synthesis. Their application has enabled precise control over phase transition [33, 34, 35], ion doping [15, 36], and the fabrication of advanced nanostructures including nanowires [37], nanorods [38], nanoplates [39], and core–shell systems [40, 41, 42].

In hot‐injection QD syntheses, suppressing MSC byproducts can narrow the size distribution of the resulting QDs. Alternatively, employing uniform MSCs as starting materials represents a complementary approach to achieving improved monodispersity [13, 43, 44, 45, 46, 47, 48], one that is particularly attractive for III–V semiconductor systems [43, 44, 47, 49]. MSCs play contrasting roles as either unwanted byproducts or beneficial precursors, which highlights the complexity of early‐stage nucleation processes. Nevertheless, the formation mechanisms underlying uniform MSCs and their interplay with QD growth during hot injection remain poorly understood, representing a key knowledge gap in the rational synthesis of monodisperse semiconductor nanocrystals.

In recent years, Yu and coworkers proposed a dual‐pathway model to elucidate the evolution of QDs and/or MSCs, delineating the transformation of precursors into intermediate precursor compounds or monomer fragments, which subsequently convert into either MSCs or QDs. Utilizing this framework, they achieved successful synthesis of CdS, CdSe, CdTe, ZnS, ZnSe, and ZnTe MSCs [16, 17, 50, 51, 52, 53, 54, 55]. Conversely, Weiss's group demonstrated that the introduction of excess surfactants or anionic ligands promotes the dissolution of CdSe MSCs [13]. The resulting monomers contribute to the sustained growth of pre‐existing clusters, ultimately leading to the formation of CdSe QDs with progressively increasing size over time. In contrast to conventional one‐step QD synthesis, this cluster‐dissolution‐mediated approach enables the production of larger nanocrystals.

**SCHEME 1:**
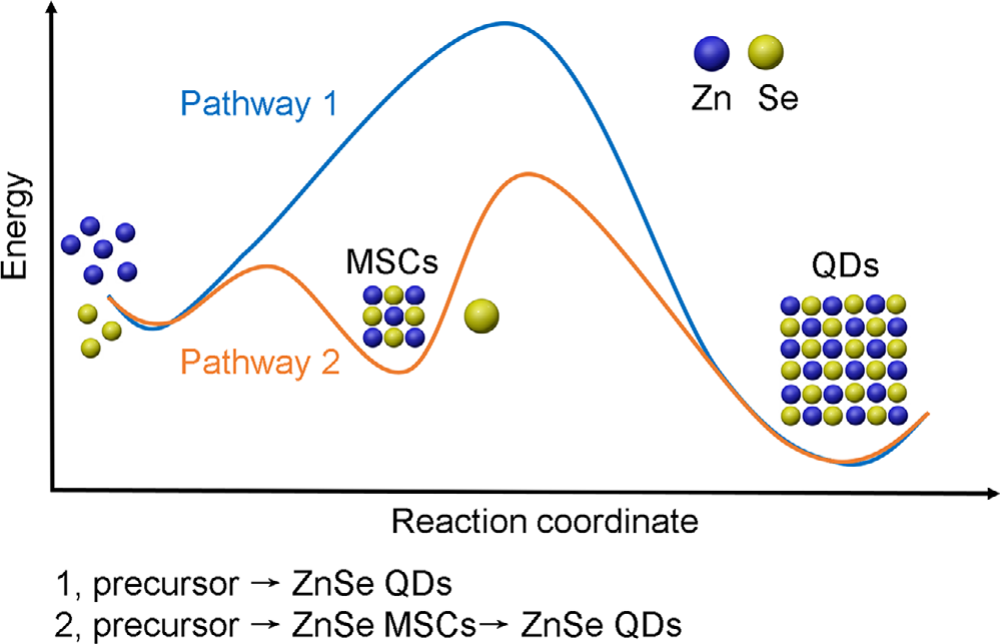
Reaction Pathways from Molecular Precursors or ZnSe MSCs to QDs.

In this work, we report the rapid, mild synthesis of a novel ZnSe MSC (denoted ZnSe MSC‐272) that exhibits exceptional thermodynamic stability. Unlike previously reported MSCs, such as CdSe clusters that dissolve into QDs upon exposure to anionic ligands [13], InP MSCs that thermally decompose into QDs at elevated temperatures [49], the ZnSe MSC‐272 clusters retained sharp and stable absorption profiles even under high‐temperature conditions and in the presence of excess anionic ligands. This observation indicates that the ZnSe MSC‐272 structure remains intact without dissolution. Transformation into QDs could only be initiated upon the introduction of additional selenium monomers (Scheme 1). In this process, Se and zinc oleate (Zn(OcA_2_)) associate with the MSCs in solution, driving a structural reorganization that yields ZnSe QDs with a final size of 6.3 nm. Although smaller than those obtained through the multi‐step injection method (e.g., 35 nm) [56], this size surpasses the typical range obtained through conventional hot‐injection synthesis, while the method itself remains straightforward. The added monomers facilitate structural reorganization and growth, offering a new mechanism for cluster‐to‐QD transitions and providing fresh insights into the interrelationship between MSCs and QDs.

## 2 Results and Discussion

### 2.1 Effects of Selenium Source and Reaction Temperature on the Formation of ZnSe MSCs

**FIGURE 1:**
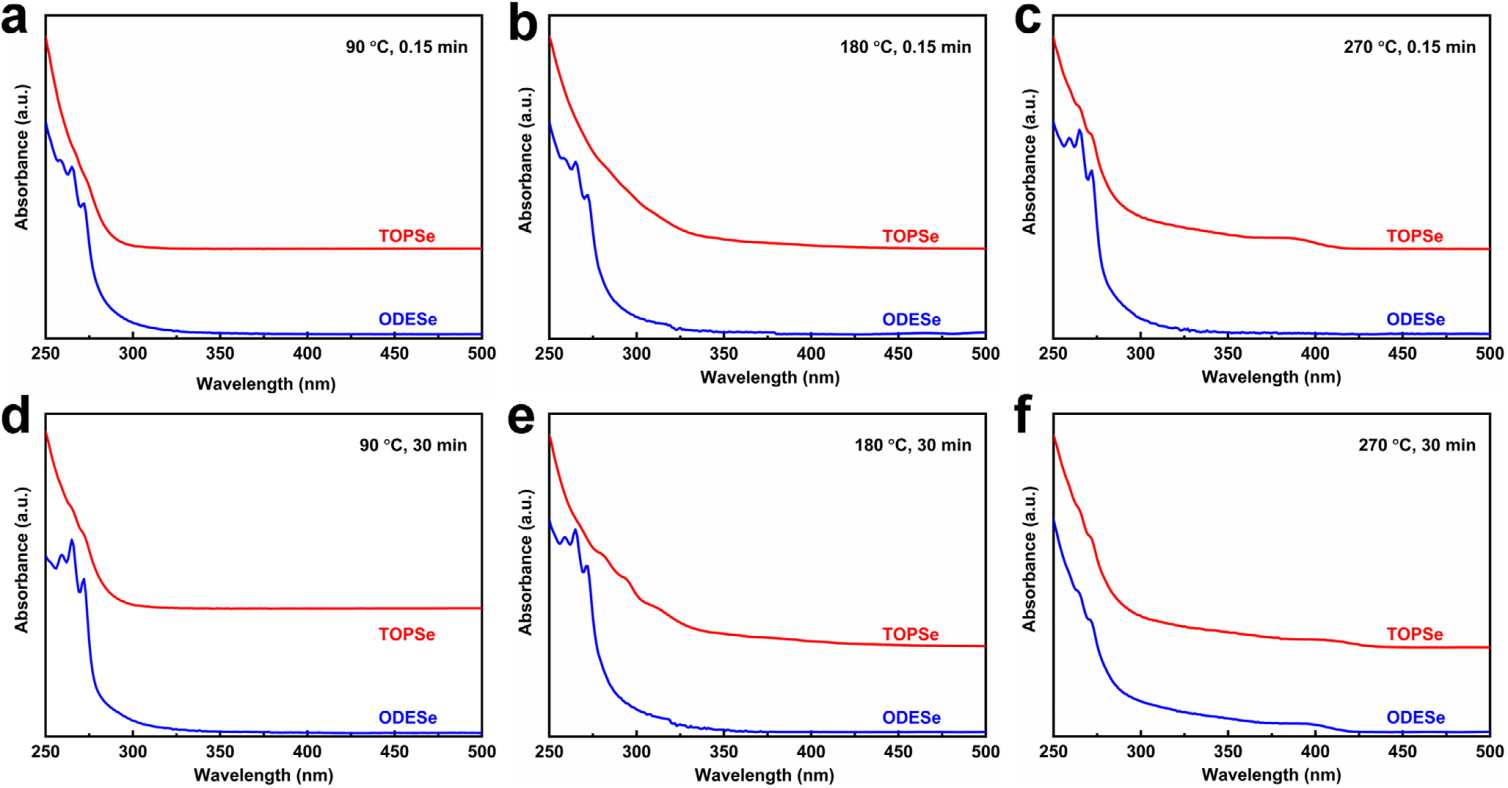
Absorption spectra of synthesized ZnSe with different Se precursors and temperatures. The spectra were recorded at 0.15 min (top) and 30 min (bottom) after injection of Se, OAM, and DPP into Zn precursors at 90°C (a, d), 180°C (b, e), and 270°C (c, f). Blue lines represent ODESe, while red lines TOPSe.

As seen in Figure 1 and Figures S1‐1, S1‐2, and S1‐3, the synthesis exhibits high phase selectivity, yielding MSC‐272 across a broad range of conditions. Specifically, within a precursor solution maintaining a Zn‐to‐octanoic acid molar ratio of 1:1, pure MSC‐272 is obtained at temperatures from 30°C to 180°C. The reactivity of the system enhances with increasing temperature, leading to an accelerated formation rate of clusters. At 90°C, both increasing and decreasing the ligand content relative to the baseline ratio still result in the formation of MSC‐272. Notably, no absorption feature corresponding to ZnSe QDs is observed throughout the entire process, confirming high phase selectivity under these conditions.

At 270°C, both MSC‐272 and ZnSe QDs are initially present, as evidenced by their respective absorption features. Over time, the MSC‐272 absorption peak gradually diminishes, while the QD absorption intensifies and progressively red‐shifts. This spectral evolution indicates a depletion of the cluster population concurrent with an increase in the average size of the QD population. These observations suggest that elevated temperatures favor the formation and growth of QDs at the expense of MSC‐272 stability. This competitive relationship is further modulated by ligand concentration. When the Zn‐to‐octanoic acid molar ratio is increased to 1:2, QD formation becomes the dominant pathway (Figure S1‐2). The excess octanoic acid ligands further suppress ZnSe MSC‐272 formation under high‐temperature conditions, reinforcing the role of ligand excess in promoting QD growth over cluster stability.

The absorption spectra also indicate that the choice of selenium source proves decisive in determining reaction outcomes. In stark contrast to the successful formation of MSC‐272 and QDs achieved with ODESe, replacement with TOPSe completely suppresses any detectable absorption features corresponding to either ZnSe MSC‐272 or QDs at both 90°C and 180°C (Figure 1; Figures S1‐1 and S1‐3). Varying the Zn‐to‐octanoic acid ratio under these conditions yields no change in this outcome. Even upon raising the temperature to 270°C and simultaneously doubling the octanoic acid content relative to zinc ions, only the characteristic absorption of ZnSe QDs emerges, with no trace of MSC formation. These results confirm that TOPSe is a compound in which selenium is stabilized via a P‐Se coordinate bond. At low temperatures, insufficient active selenium monomers are available, and TOP molecules act as ligands coordinating with zinc to reduce the concentration of free zinc ions. This dual effect suppresses the nucleation of MSCs. At high temperatures, TOPSe undergoes slow thermal decomposition to release active selenium monomers, which fail to reach the high supersaturation required for MSC nucleation. Meanwhile, the concentration of active zinc in the system increases, driving the reaction directly toward QD growth.

### 2.2 Effects of Ligands on the Formation of ZnSe MSCs

**FIGURE 2:**
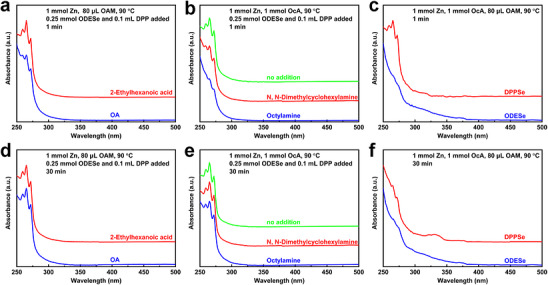
Absorption spectra of synthesized ZnSe with different ligands. The spectra were recorded at 1 min (top) and 30 min (bottom) after injection of Se into the Zn precursors at 90°C. In (a, d), blue lines represent OA, while red lines stand for 2‐Ethylhexanoic acid. In (b, e), blue lines represent n‐Octylamine, red lines DMCHA, and green lines represent no amine. In (c, f), blue lines represent ODESe, while red lines represent DPPSe.

Substituting octanoic acid (OcA) with long‐chain oleic acid (OA) or branched‐chain 2‐ethylhexanoic acid does not alter the reaction outcome. In all cases, absorption spectra (Figure 2; Figure S2‐1) reveal the formation of ZnSe MSC‐272 within just 0.15 min of reaction, with no significant spectral evolution observed upon extending the reaction time to 30 min. These results demonstrate that the type of carboxylic acid ligand (varying in chain length or branching) exerts only a weak influence on the formation kinetics or stability of ZnSe MSC‐272.To assess the role of amine ligands, we investigated their impact on ZnSe MSC formation using amines varying in chain length and steric profile. As shown in the absorption spectra (Figure 2; Figure S2‐2), sharp characteristic MSC absorption peaks appear within 0.5 min of reaction, irrespective of whether short‐chain linear primary amines (e.g., octylamine), branched tertiary amines (e.g., DMCHA), or no amine ligands are present. Furthermore, extending the reaction time to 30 min produces no significant evolution in the absorption features. These observations collectively indicate that the presence, absence, or structural variation of amine ligands likewise has only a minor effect on the formation kinetics or stability of ZnSe MSC‐272.

To elucidate the role of the activating agent DPP in the formation of ZnSe MSC‐272, three sets of experiments with varying DPP concentrations were conducted at 90°C. Absorption spectra collected within the first 0.5 min of reaction (Figure 2; Figure S2‐3) reveal that both the ODESe + DPP system and the pre‐synthesized DPPSe (1 m) system support the formation of MSC‐272, as evidenced by a well‐defined absorption peak. However, by the 1‐minute mark, the characteristic peaks at 272 nm and 265 nm in the DPPSe system begin to diminish, indicating gradual decomposition of the initially formed clusters. Concurrently, a new absorption feature emerges at 331 nm and remains stable thereafter. In contrast, in the absence of DPP, no characteristic absorption at either 272 nm or 331 nm is observed throughout the reaction. These results demonstrate that an appropriate concentration of DPP is essential for the formation of ZnSe MSC‐272. However, directly substituting DPPSe for the ODESe + DPP system compromises cluster stability, leading to their decomposition and the formation of a distinct, stable species absorbing at 331 nm.

As presented in Figure S2‐4, ^1^H NMR and ^3^ ^1^P NMR were measured to further reveal the role of DPP in the reaction mechanism. When ODESe or ODESe + DPP was introduced into a Zn(OcA)_2_ solution and allowed to react for 30 min, the triplet peak of Zn(OcA)_2_ at 2.34 ppm shifted to 2.30 ppm and 2.31 ppm, respectively, suggesting the formation of ZnSe precursor complexes or ZnSe MSCs. In contrast, no such shifts were observed upon addition of TOPSe + DPP or DPPSe, indicating the absence of analogous ZnSe species under these conditions. As shown in Figure S2‐4b, the ^3^ ^1^P NMR signal of DPP, originally a singlet at 21.53 ppm, shifted to 22.14 ppm and 22.07 ppm after reaction with selenium to form DPPSe and after introduction to the zinc precursor solution, respectively. Notably, when DPPSe or ODESe + DPP was added to the zinc precursor solution, the ^3^ ^1^P resonance returned to approximately 21.53 ppm. This chemical shift recovery indicates the dissociation of selenium from DPP, followed by the formation of Zn─Se bonds, thus underscoring the role of DPP as a reversible selenium‐activating agent in the nucleation process of ZnSe MSCs. When TOPSe + DPP is introduced, the signal corresponding to DPP completely disappears, and the newly appeared signal is a complex multiplet centered around 32 ppm, characteristic of a diphosphine selenide species. Consequently, no zinc selenide clusters or related compounds are formed. This conclusion is further supported by ^1^H‐NMR spectroscopy, where no characteristic signals of zinc selenide‐carboxylate species are observed.

### 2.3 Stability of ZnSe MSCs

**FIGURE 3:**
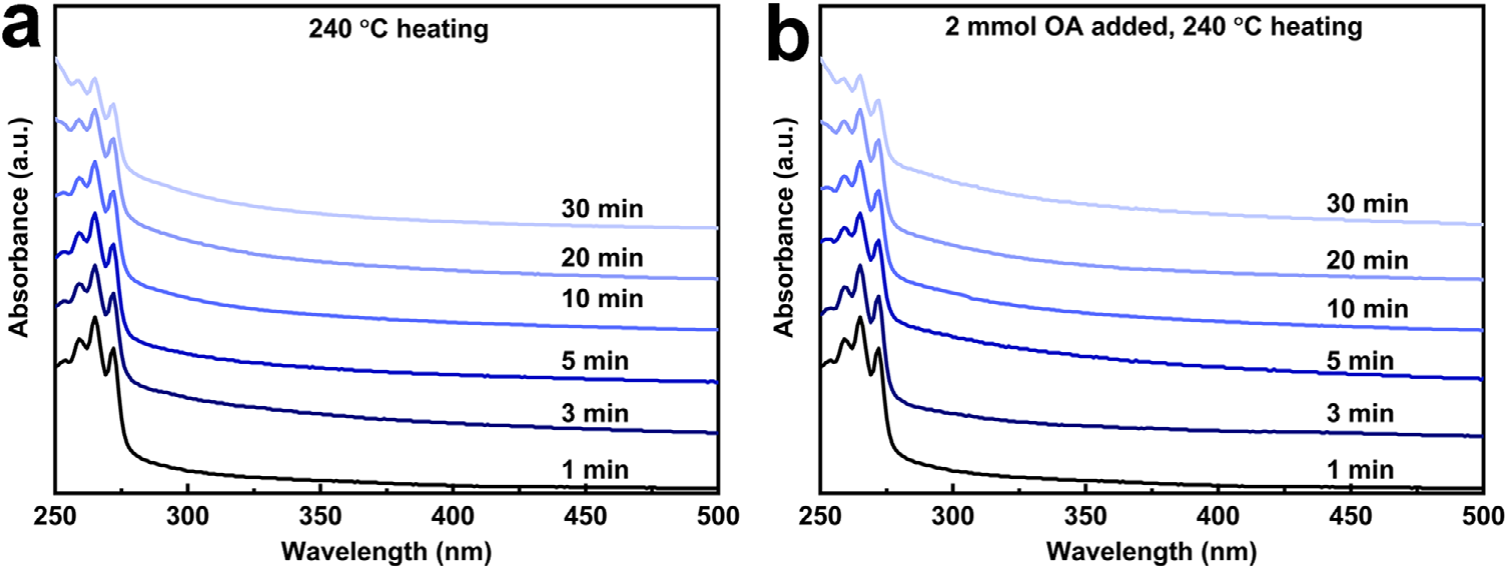
Time‐dependent absorption spectra of ZnSe MSC‐272 solution following thermal treatment at 240°C (a) without and (b) with the addition of 2 mmol OA ligand.

When ODESe and DPP are added directly to the zinc precursor solution at elevated temperatures (210–300°C), absorption spectra (Figure 1; Figure S3) reveal the concurrent formation of both MSCs and QDs, indicating a competitive nucleation pathway. As the temperature increases, QD formation becomes increasingly dominant, occurring more rapidly and yielding larger particles. In stark contrast, when ZnSe MSC‐272 is first synthesized at 90°C and subsequently subjected to the same elevated temperatures (210–300°C), the sharp characteristic absorption peaks remain unchanged (Figure 3; Figure S3), demonstrating exceptional thermal stability. Up to 240°C, only MSC‐272 absorption is observed, with no detectable band‐edge absorption from QDs, indicating that thermal treatment alone does not induce transformation of pre‐formed MSCs into QDs within this temperature range. Furthermore, even after the introduction of excess OA, the MSC‐272 absorption profile persists without any sign of ZnSe QD formation, confirming its high stability under both thermal and ligand‐rich conditions.

### 2.4 Conversion of ZnSe MSCs to ZnSe QDs

When synthesis is conducted directly at elevated temperatures, ZnSe MSC‐272 and QDs form simultaneously via competitive nucleation. In contrast, pre‐formed MSC‐272 synthesized at low temperatures cannot be converted into QDs by thermal treatment alone or by the introduction of excess OA, demonstrating exceptional acidothermal stability. This resilience suggests the presence of a protective kinetic barrier surrounding the pre‐assembled clusters, which inhibits their transformation under otherwise favorable conditions. However, as shown in Figure 1 and Figure S4‐1, the introduction of either ODESe or TOPSe into a solution of ZnSe MSC‐272 at 180 °C triggers a noticeable attenuation of the characteristic MSC absorption peak, indicating a reduction in cluster concentration and the onset of transformation into QDs. These results reveal that while MSC‐272 is inherently stable against heat and ligand‐rich environments, the addition of exogenous selenium monomers is sufficient to overcome the kinetic barrier and initiate the cluster‐to‐QD transition.

**FIGURE 4:**
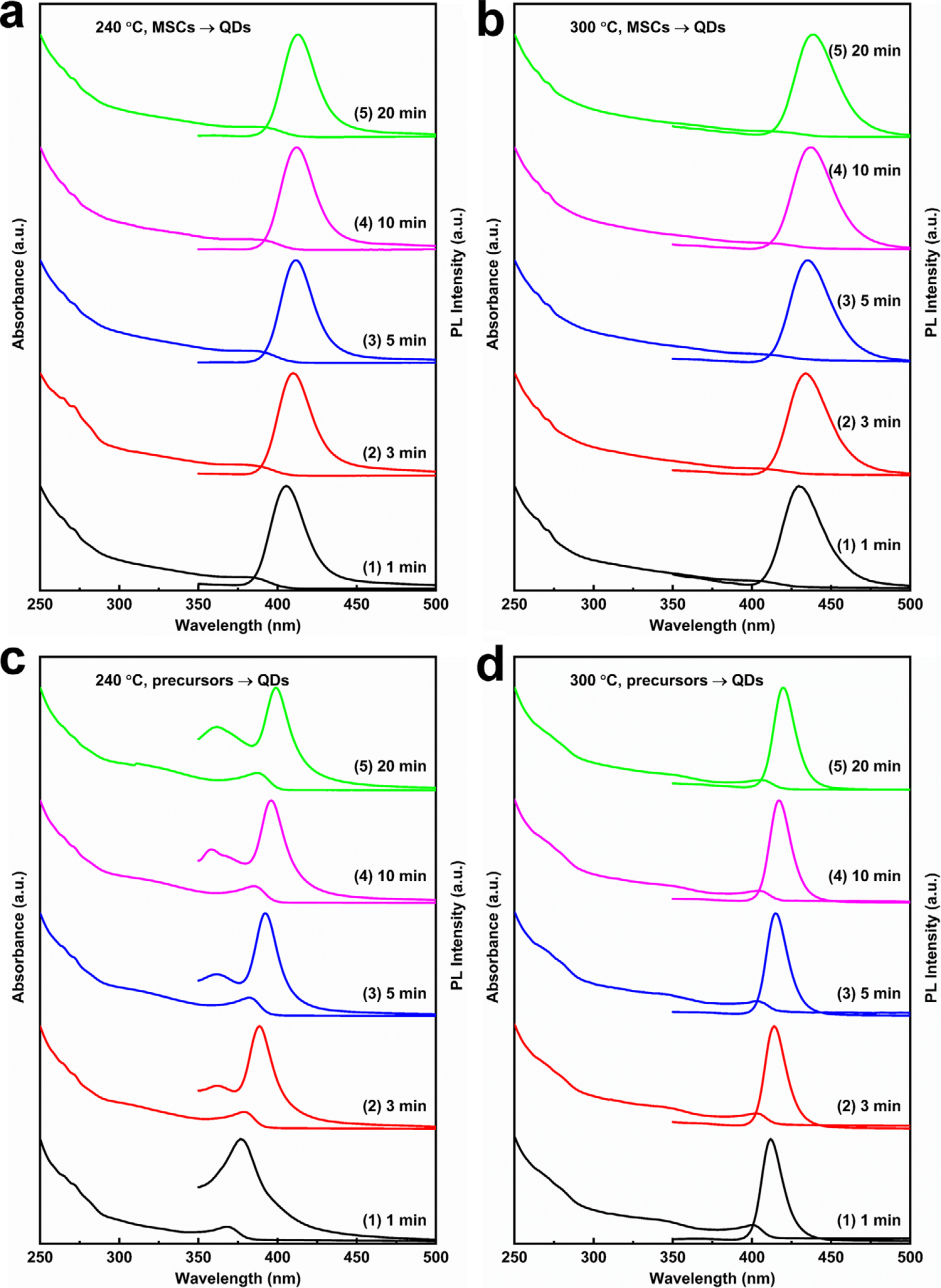
Synthesis of ZnSe QDs through different methods. Evolution of the UV–vis absorption and PL spectra of ZnSe QDs fabricated from MSCs at (a) 240°C and (b) 300°C, and from molecular precursors at (c) 240°C and (d) 300°C.

Additionally, the first excitonic absorption peak reveals a continuous redshift of the first excitonic peak over the course of the reaction (Figures 1 and 4), consistent with the quantum confinement effect characteristic of QD growth. This spectral evolution confirms that the kinetic barrier protecting ZnSe MSC‐272 has been surmounted, enabling cluster transformation and subsequent QD formation. The rate of this redshift and thus the conversion rate of MSC‐272 to QDs is sensitive to reaction conditions. Specifically, elevated temperatures or the addition of excess OA (Figure S4‐1) accelerate the redshift, reflecting faster cluster‐to‐QD conversion. These conditions also yield larger QDs, likely due to enhanced dissolution of zinc species and accelerated Ostwald ripening, both promoted by higher temperatures and ligand‐rich environments. High‐resolution transmission electron microscopy (HRTEM) images of ZnSe MSC‐272 and the resulting ZnSe QDs are provided in Figures S4‐2 and S4‐3, respectively. As shown in Figure S4‐4, X‐ray diffraction (XRD) analysis confirms that all ZnSe QDs derived from MSCs exhibit the zinc blende crystal structure.

Notably, QDs derived from MSCs consistently exhibited larger sizes (as evidenced by their longer‐wavelength absorption shown in Figure 4 and the TEM images in Figure S4‐5), compared to those from conventional precursors at identical reaction temperatures. The photoluminescence (PL) of MSC‐derived QDs exhibited a systematic redshift from 384 nm to 439 nm as the synthesis temperature increased from 180°C to 300°C, QDs with good size distribution (FWHM = 24.2–25.8 nm) are achieved over a wide temperature range of 210–270°C (Figure 4; Figure S4‐6). In contrast, QDs synthesized via the conventional precursor monomer route under identical conditions (300°C, 20 min) displayed a PL peak at 420 nm, corresponding to a measured size of 4.3 nm, notably smaller than the 6.3 nm obtained from the MSC‐mediated route at the same temperature. Furthermore, the conventional route yielded a PLQY of 2.02%, a FWHM of 16 nm, and a lifetime of 12.39 ns, whereas the MSC‐derived QDs showed a PLQY of 4.33%, a FWHM of 30 nm, and a lifetime of 16.85 ns (Figure S4‐7). The broader FWHM in the MSC‐derived case is attributed to partial changes in surface ions at 300°C (as suggested by the absorption spectrum in Figure S3), which degrade compositional and size uniformity, leading to a broader size distribution. XRD analysis confirms that both types of QDs adopt the zinc blende crystal structure (Figure S4‐8), indicating that the observed size discrepancy arises from distinct growth pathways rather than differences in crystalline phase. We propose that pre‐formed MSCs serve as stable growth seeds, effectively suppressing continuous nucleation and promoting focused growth under a controlled monomer supply. This mechanism not only rationalizes the observed larger particle size but also highlights the potential of the cluster‐mediated route as a robust strategy for achieving size‐controlled synthesis of high‐quality QDs, particularly in the larger size regime that is challenging for conventional methods.

## 3 Conclusion

In summary, this work presents a rapid synthesis of novel ZnSe MSCs under mild conditions and demonstrates a cluster‐mediated pathway for the preparation of ZnSe QDs through supplemental selenium introduction. The influence of critical parameters, including injection temperature, ligand identity and concentration, selenium source, and activating agents, on the formation of highly stable ZnSe MSCs. The exceptional stability of the resulting MSCs highlights their distinct structural nature even under high‐temperature and ligand‐rich environments. Furthermore, a cluster‐mediated conversion pathway to ZnSe QDs was realized, providing mechanistic insight into the competitive nucleation processes between MSCs and QDs and offering enhanced regulatory precision in large‐size nanocrystal synthesis.

## Conflicts of Interest

The authors declare no conflict of interest.

## Supporting information




**Supporting File**: advs75439‐sup‐0001‐SuppMat.docx.

## Data Availability

The data that support the findings of this study are available from the corresponding author upon reasonable request.
