# Supplementary material for: Synthesis of Novel Stable ZnSe Magic‐Sized Clusters and Their Direct Transformation to Quantum Dots
Source: Adv Sci (Weinh). 2026 May 4;13(40):e75439. doi: 10.1002/advs.75439 (PMC13335569; doi:10.1002/advs.75439)
Supplement: Supplementary file 1 — Supporting File: advs75439‐sup‐0001‐SuppMat.docx. [file ADVS-13-e75439-s001.docx]

**Supporting Information**

**Synthesis of Novel Stable ZnSe Magic-Sized Clusters and Their Direct Transformation to Quantum Dots**

Bin Song,^[a]^ Zihui Jin,^[b]^ Xuanyu Zhang,^[c]^ and Rong-Jun Xie*^[a]^

[a] B. Song, R. Xie

Fujian Key Laboratory of Surface and Interface Engineering for High Performance Materials

College of Materials, Xiamen University

Xiamen 361005, P. R. China

E-mail: rjxie@xmu.edu.cn

[b] Z. Jin

College of Materials Science and Engineering, Science and Education Integration College of Energy and Carbon Neutralization, Zhejiang University of Technology

Hangzhou 310014, P. R. China.

[c] X. Zhang

School of Physics and Optoelectronic Engineering, Hangzhou Institute for Advanced Study, University of Chinese Academy of Sciences

Hangzhou 310014, P. R. China.

*Corresponding Author.

E-mail: [rjxie@xmu.edu.cn](mailto:rjxie@xmu.edu.cn);

**Experimental Methods**

**Chemicals**

Zinc acetate (Zn(OAc)_2_, 99.99%), oleic acid (OA, 90%), n-Octanoic acid (OcA, 99%), 2-Ethylhexanoic acid (99%), oleylamine (OAM, 70%), n-Octylamine(99%), N,N-Dimethylcycloohexylamine (DMCHA, 98%), diphenylphosphine (DPP, 95%), 1-octadecene (ODE, 90%), hexane (98.5%) and ethanol (99.9%) were purchased from Aladdin. Selenium powder (Se, 99.999%), 1-octadecene (ODE, 90%), tri-n-octylphosphine (TOP, 90%), and chloroform-d (CDCl_3_ , 99.8%) were purchased from Sigma-Aldrich. All chemicals were used as received without further purification unless other wise stated.

**Preparation of Zn(OcA)_2_, Zn(OA)_2_, Zinc 2-ethylhexanoate, ODESe, TOPSe and DPPSe Stock Solutions**

For Zinc octanoate (Zn(OcA)_2_) stock solution, Zn(OAc)_2_ (0.9174 g, 5.00 mmol), OcA (0.7210 g, 5 mmol) and ODE (49.22 mL) were loaded in a 100 mL three-necked flask. The mixture was stirred and degassed and backfilled with argon (Ar) at room temperature. This procedure was repeated three times. Under an Ar atmosphere, the mixture was then heated to 160 °C until the precursors were completely dissolved. The resulting solution was cooled to room temperature and stored in the glove box for further use.

For Zinc oleate (Zn(OA)_2_) stock solution, Zn(OAc)_2_ (0.9174 g, 5.00 mmol), OA (1.412 g, 5 mmol) and ODE (48.42 mL) were loaded in a 100 mL three-necked flask. The mixture was treated same with Zn(OcA)_2_. The resulting solution was cooled to room temperature and stored in the glove box .

For Zinc 2-ethylhexanoate stock solution, Zn(OAc)_2_ (0.9174 g, 5.00 mmol), 2-Ethylhexanoic acid (0.7210 g, 5 mmol) and ODE (49.2 mL) were loaded in a 100 mL three-necked flask. The mixture was treated same with Zn(OcA)_2_. The resulting solution was cooled to room temperature and stored in the glove box .

For ODESe stock solution, Se powder (0.1579 g, 2.00 mmol) and ODE (10 mL) were loaded in a 50 mL three-necked flask. The mixture was stirred and degassed and backfilled with Ar at room temperature. This procedure was repeated three times. Under a Ar atmosphere, the mixture was then heated to 260 °C and stirred at this temperature for 4 h. The resulting solution was cooled to room temperature. The prepared ODESe stock solution was stored in the glove box.

For TOPSe stock solution, Se powder (0.7896 g, 10.00 mmol) and TOP (10 mL) were loaded in a 50 mL three-necked flask. The mixture was heated to 120 °C and stirred at this temperature for 30 min in the glove box. The resulting solution was cooled to room temperature and stored in the glove box.

For DPPSe stock solution, Se powder (0.7896 g, 10.00 mmol) and DPP (10 mL) were loaded in a 50 mL three-necked flask. The mixture was heated to 120 °C and stirred at this temperature for 30 min in the glove box. The resulting solution was cooled to room temperature and stored in the glove box.

**Synthesis of ZnSe MSCs**

10 mL Zn(OcA)_2_ stock solution (Zn = 1.00 mmol) was placed in a 50 mL three-neck flask. The solution was heated up to 90 °C, then a mixture of the ODESe stock solution (1.25 mL, Se = 0.25 mmol), OAM (0.0669 g, 0.25 mmol) and DPP (0.0931 g, 0.50 mmol) were added. Eight samples were extracted from 0.15 to 30 min. Then the reaction solution was naturally cooled down to room temperature. The ZnSe MSCs solution was mixed with 10 mL of hexane and 20 mL of ethanol, followed by centrifugation. The precipitate was retained, redissolved in 5 mL of hexane, and then subjected to another centrifugation after the addition of 10 mL of ethanol. The final precipitate was dissolved in hexane.

**The thermal stability of ZnSe MSCs**

10 mL solution of ZnSe MSCs (Zn = 1.00 mmol) synthesized at 90 °C, was heated from 210 °C to 300 °C in 30 °C increments, then six samples were extracted from 1 to 30 min.

10 mL solution of ZnSe MSCs (Zn = 1.00 mmol) synthesized at 90 °C, was heated to 240 °C, then 1 mmol OA (0.2825 g) was added, six samples were extracted from 1 to 30 min.

**Synthesis of ZnSe quantum dots from MSCs**

10 mL solution of ZnSe MSCs (Zn = 1.00 mmol) synthesized at 90 °C, was heated from 180 °C to 300 °C in 30 °C increments, then 1.8 mL TOPSe (Se=0.36 mmol) stock solution was added. Five samples were extracted from 1 to 20 min.

10 mL solution of ZnSe MSCs (Zn = 1.00 mmol) synthesized at 90 °C, was heated to 240 °C, then 1.8 mL TOPSe (Se=0.36 mmol) stock solution and OA (0.2825 g, 1 mmol) were added. Five samples were extracted from 1 to 20 min.

10 mL solution of ZnSe MSCs (Zn = 1.00 mmol) synthesized at 90 °C, was heated from 180 °C or 240 °C, then 1.8 mL ODESe (Se=0.36 mmol) stock solution was added. Five samples were extracted from 1 to 20 min.

10 mL solution of ZnSe MSCs (Zn = 1.00 mmol) synthesized at 90 °C, was heated to 240 °C, then 1.8 mL ODESe (Se=0.36 mmol) stock solution and OA (0.2825 g, 1 mmol) were added. Five samples were extracted from 1 to 20 min.

**Synthesis of ZnSe quantum dots from precursors**

10 mL Zn(OA)_2_ stock solution (Zn = 1.00 mmol) was placed in a 50 mL three-neck flask. The solution was heated up to 240 °C or 300 °C, then a mixture of the TOPSe stock solution (0.6 mL, Se = 0.6 mmol), OAM (0.0669 g, 0.25 mmol) and DPP (0.0931 g, 0.50 mmol) were added. Five samples were extracted from 1 to 20 min. The mixture was then quenched to room temperature. The ZnSe QDs solution was mixed with 10 mL of hexane and 20 mL of ethanol, followed by centrifugation. The precipitate was retained, redissolved in 5 mL of hexane, and then subjected to another centrifugation after the addition of 10 mL of ethanol. The final precipitate was dissolved in hexane.

**Characterization**

Absorption spectra of the samples were recorded using PerkinElmer Lambda 1050 UV/Vis spectrophotometer. Steady-state PL spectra were measured using the Hitachi F-4600. Photoluminescence quantum yield was obtained by Hamamatsu C13347. Time-resolved PL decay spectra of QDs were obtained by Edinburgh Instruments FLS980. HRTEM of MSCs and QDs were obtained by a Talos F200X with 200 kV acceleration voltage. For the analysis of ^1^H-NMR and ^31^P-NMR, MSCs were dissolved in Chloroform-d and the signals were recorded using a Bruker AVANCE NEO 500 spectrometer. The XRD patterns of the QDs were characterized by Bruker D8 Advanced Davinci using Cu Kα radiation (λ = 1.5418 Å).


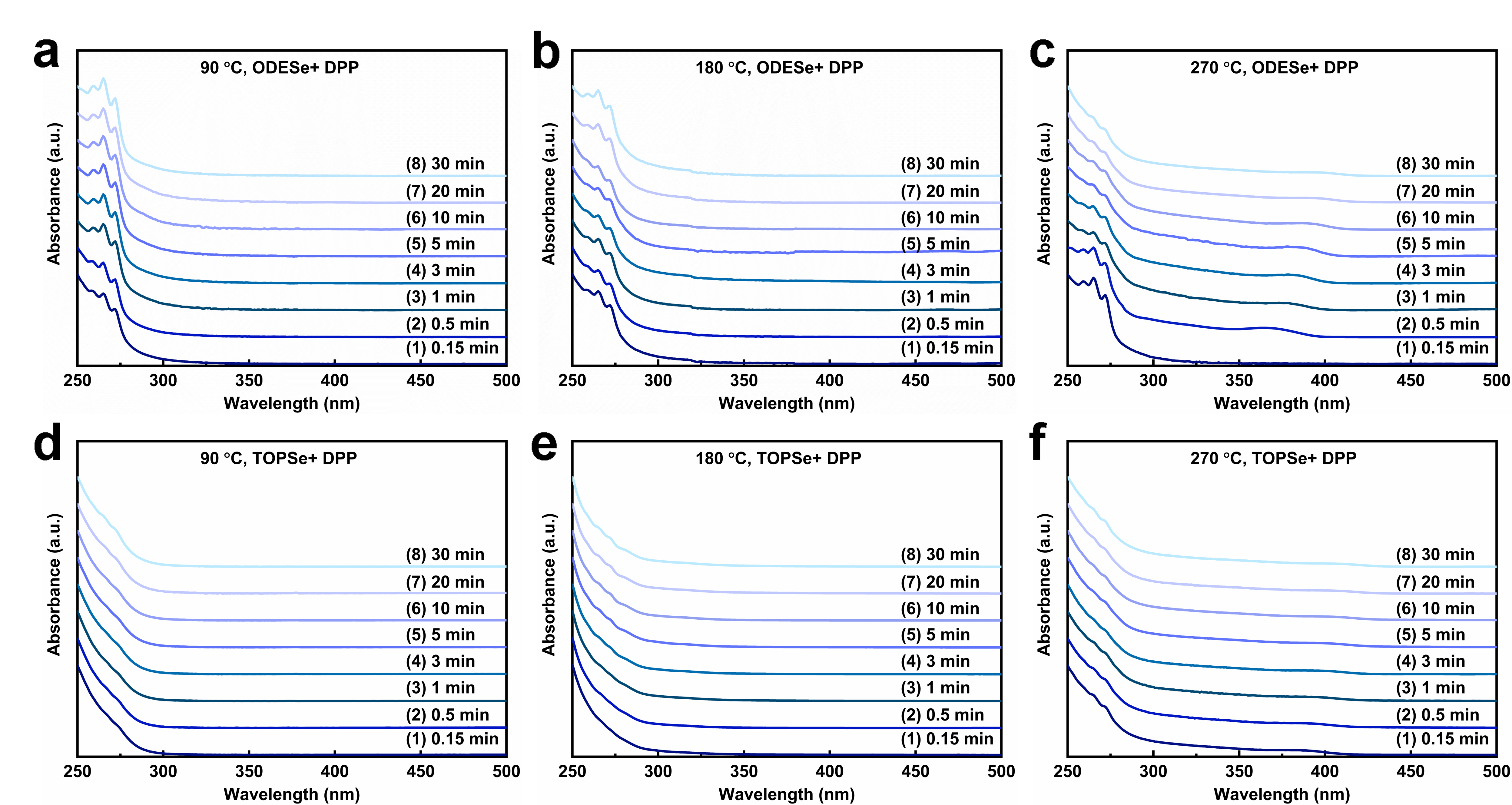


Figure S1-1, Absorption spectra of synthesized ZnSe obtained under different Se precursors and reaction temperatures. Panels (a-c) correspond to injections of OAM, DPP, and ODESe into the Zn precursors at 90 °C, 180 °C, and 270 °C, respectively. Similarly, panels (d-f) show the results when TOPSe replaces ODESe, injected at 90 °C, 180 °C, and 270 °C.


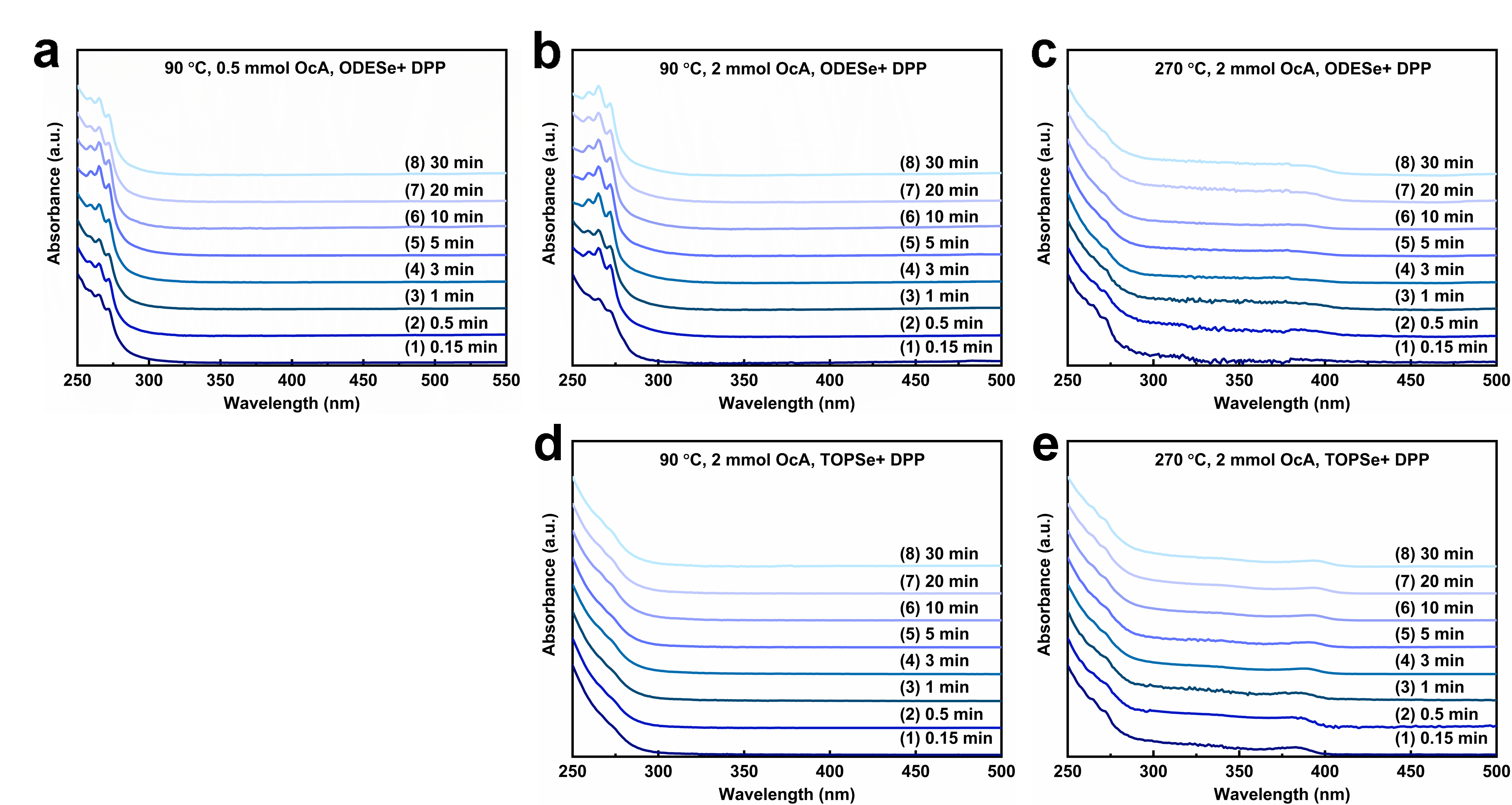


Figure S1-2, Absorption spectra of ZnSe synthesized under systematically varied conditions. (a-c) Using ODESe/DPP with OcA: (a) 0.5 mmol OcA at 90 °C, (b) 2 mmol OcA at 90 °C, (c) 2 mmol OcA at 270 °C. (d, e) Using TOPSe with OcA: (d) 2 mmol OcA at 90 °C, (e) 2 mmol OcA at 270 °C.


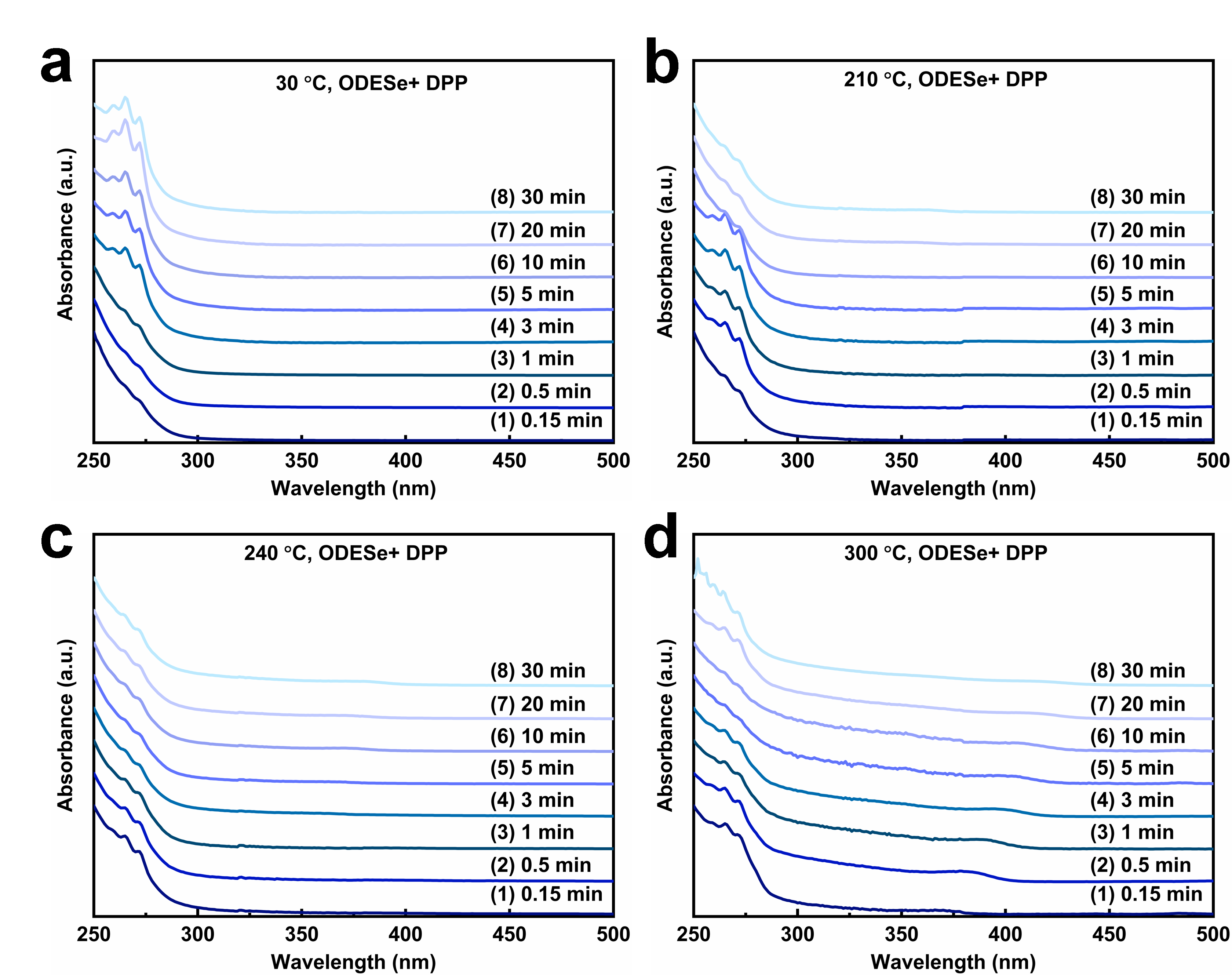


Figure S1-3, Absorption spectra of ZnSe synthesized with ODESe/DPP at different temperatures: (a) 30 °C, (b) 210 °C, (c) 240 °C, (d) 300 °C.


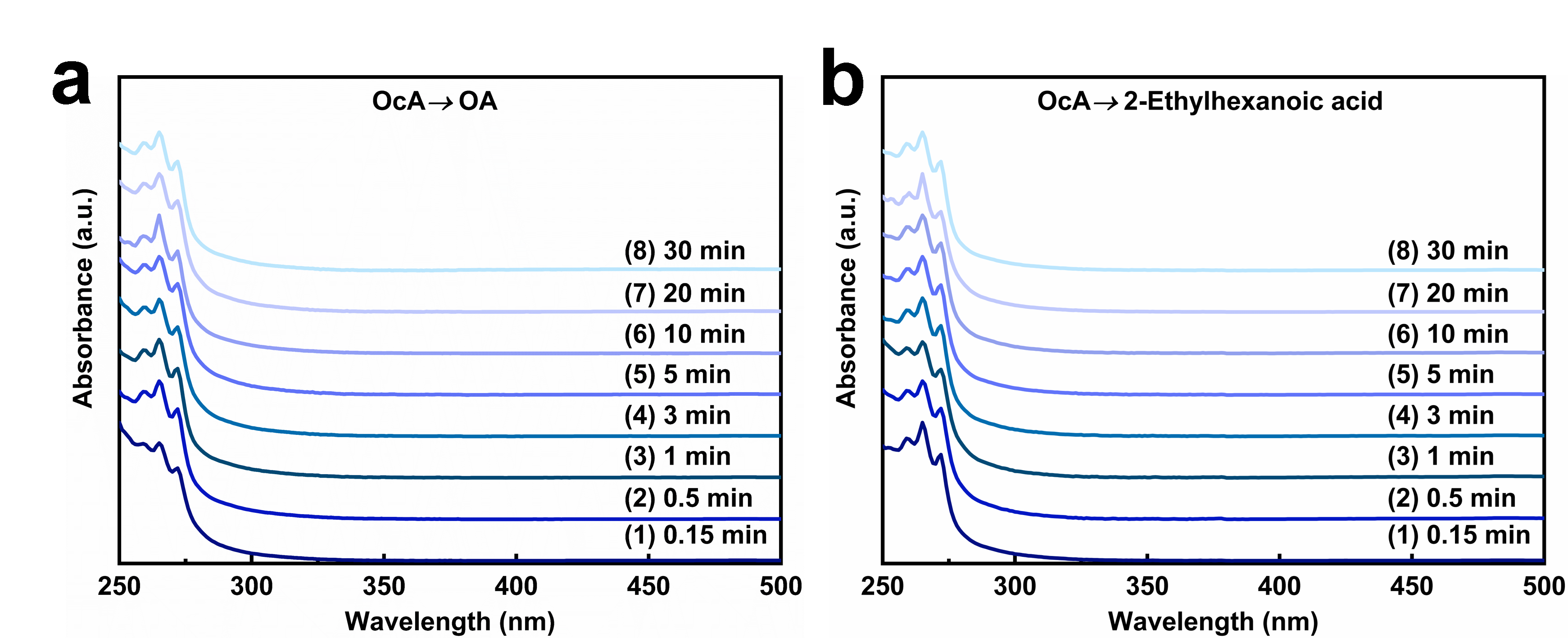


Figure S2-1, Absorption spectra of synthesized ZnSe with different acid ligands. (a) OA, (b) 2-Ethylhexanoic acid.


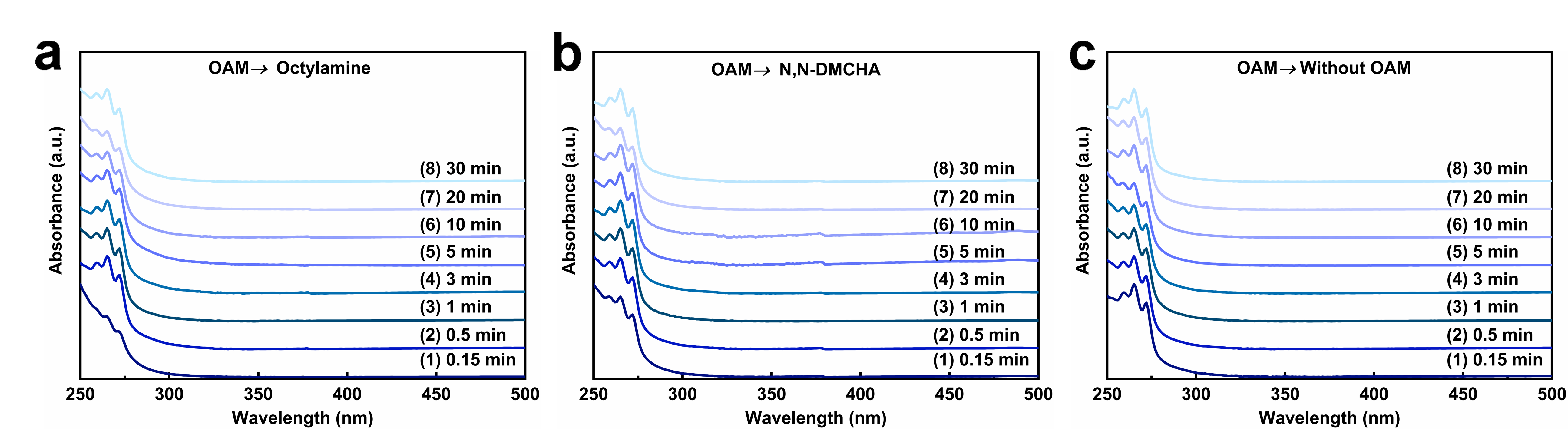


Figure S2-2, Absorption spectra of synthesized ZnSe with different amine ligands. (a) n-Octylamine, (b) DMCHA, (c) control experiment without amine.


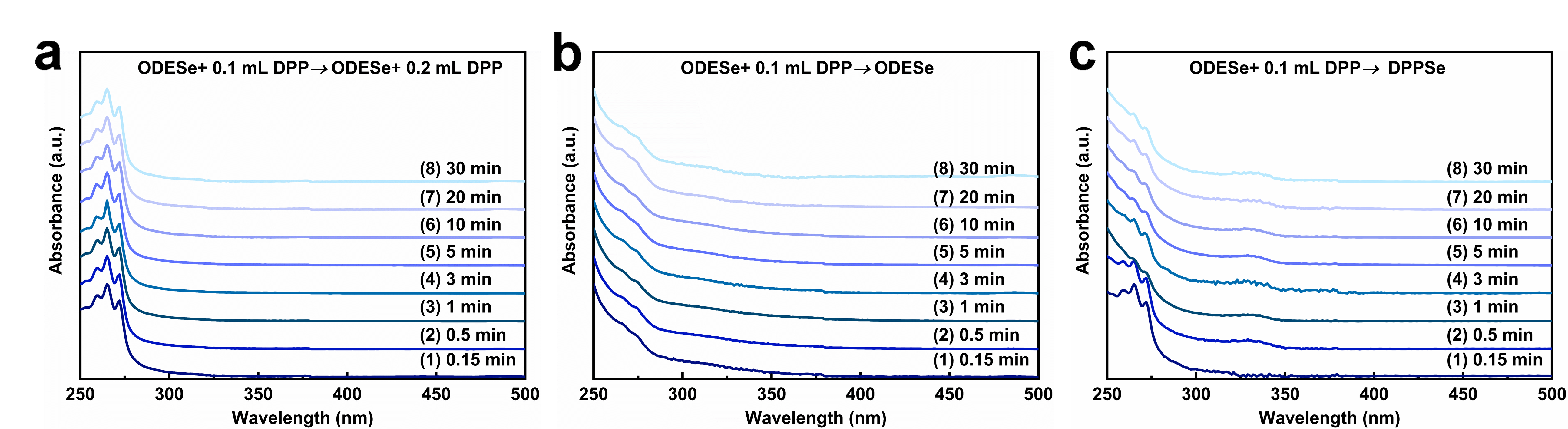


Figure S2-3, Absorption spectra of ZnSe synthesized with different DPP loadings. (a) 0.2 mL DPP, (b) No DPP, (c) DPPSe precursor (Se content: 0.25 mmol).


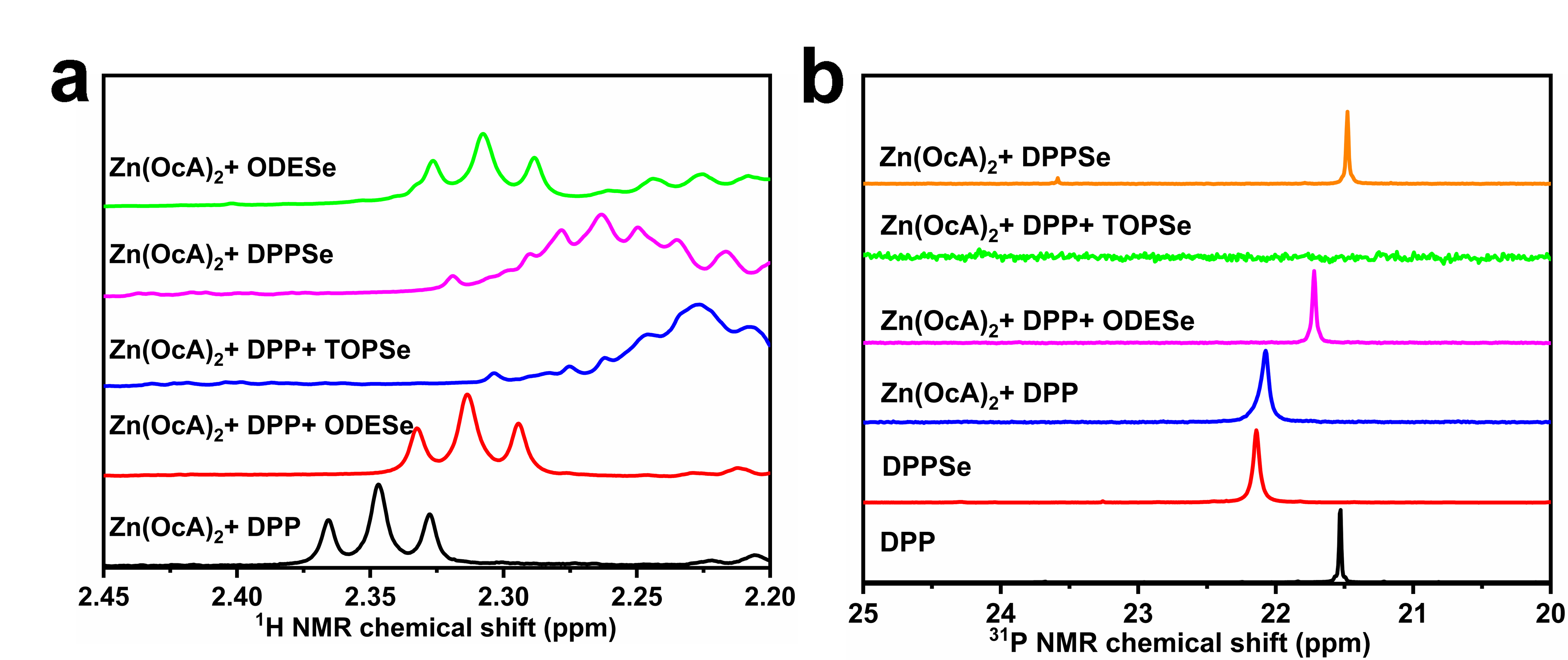


Figure S2-4, Comparative NMR analysis: (a) ¹H-NMR and (b) ³¹P-NMR spectra of ZnSe and their precursors.


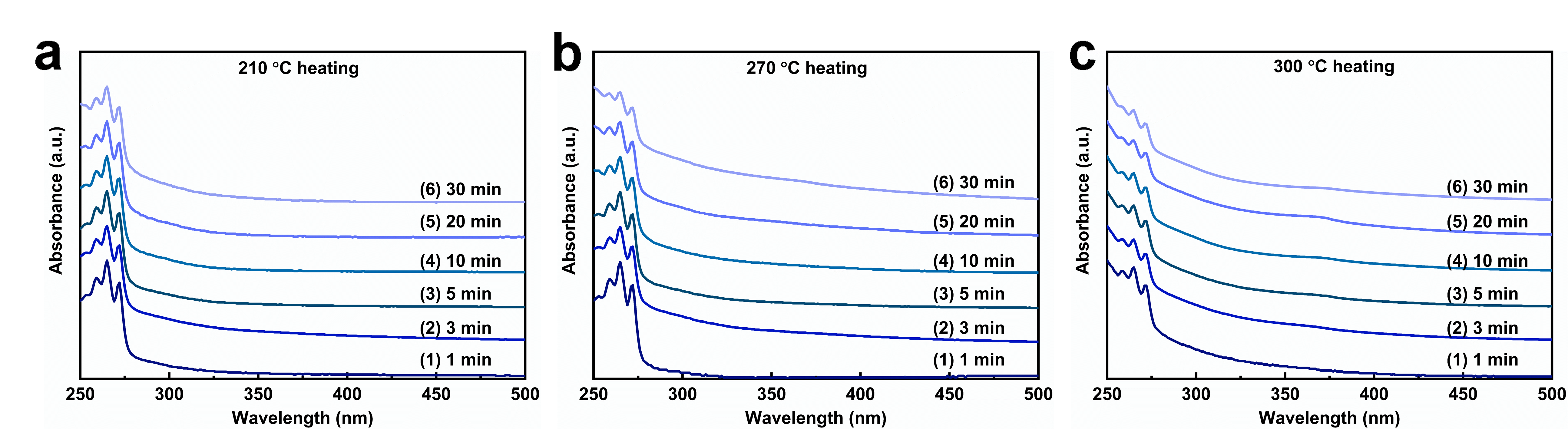


Figure S3, Time-dependent absorption spectra of ZnSe MSC-272 solution after thermal treatment at: (a) 210 °C, (b) 270 °C, (c) 300 °C.


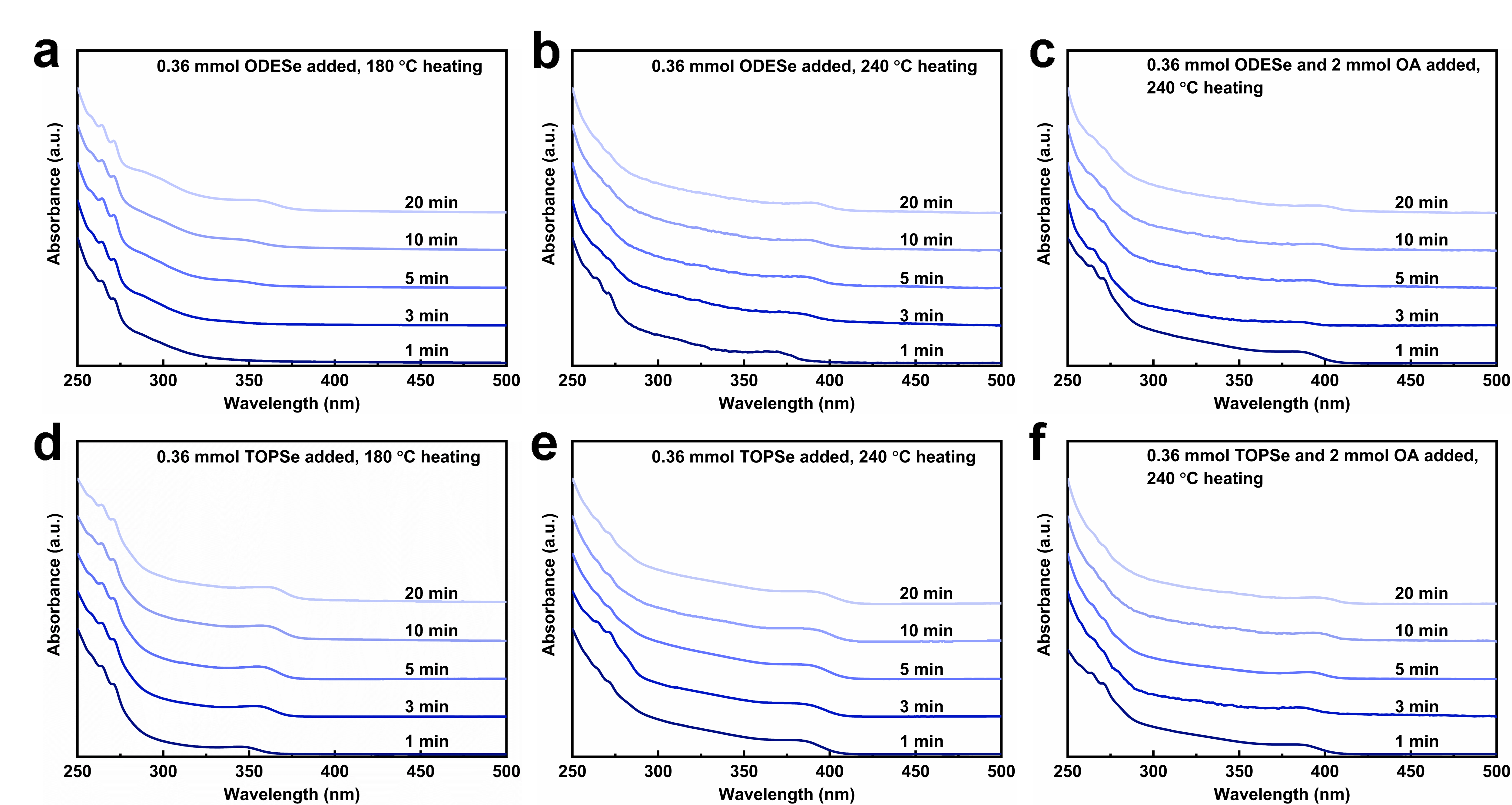


Figure S4-1, Absorption spectra of ZnSe quantum dots synthesized under varied conditions. Using ODESe as the selenium source: (a) 180 °C, (b) 240 °C, (c) 240 °C with extra OA. Using TOPSe as the selenium source: (d) 180 °C, (e) 240 °C, (f) 240 °C with extra OA.


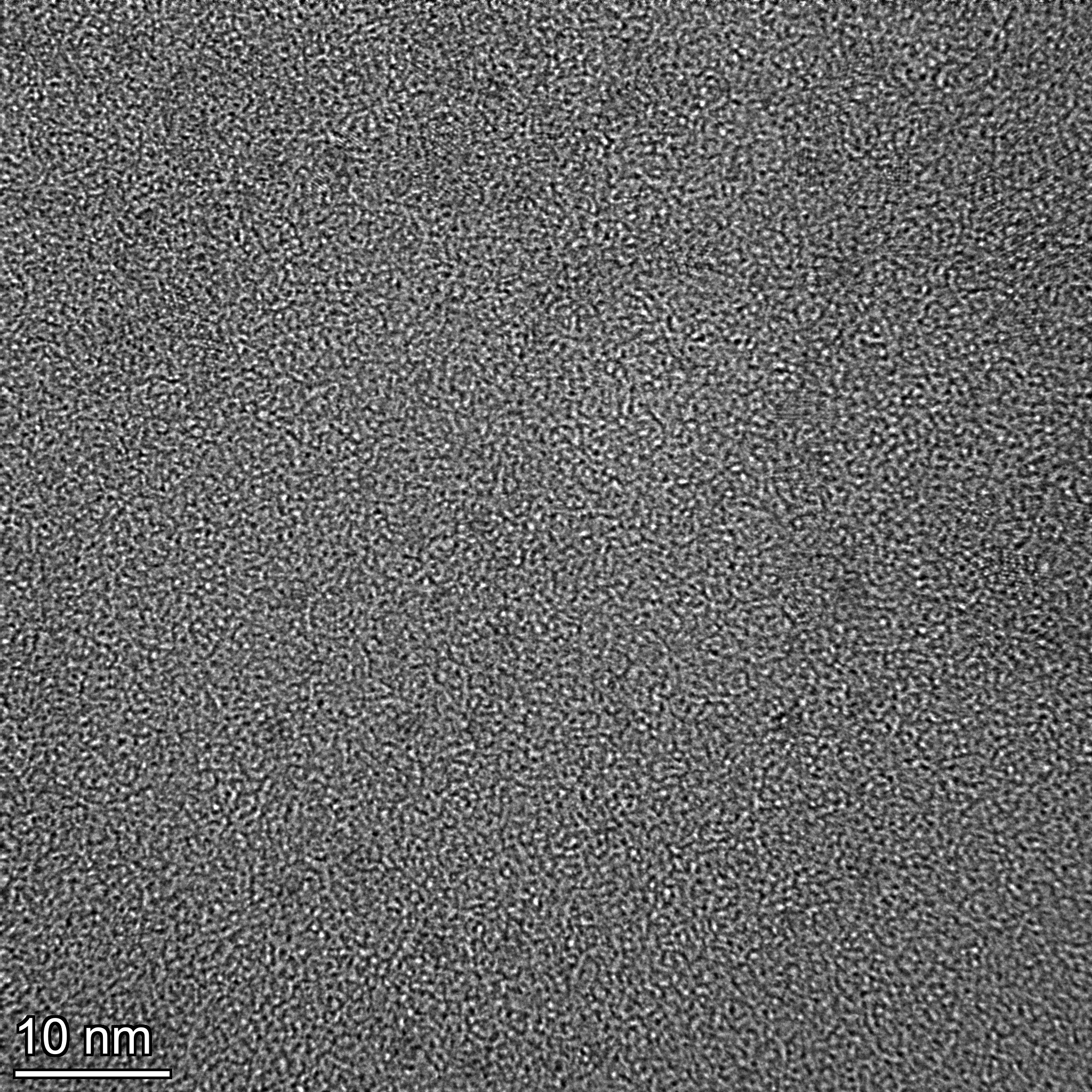


Figure S4-2, High-resolution transmission electron microscope (HRTEM) image of ZnSe MSC-272.


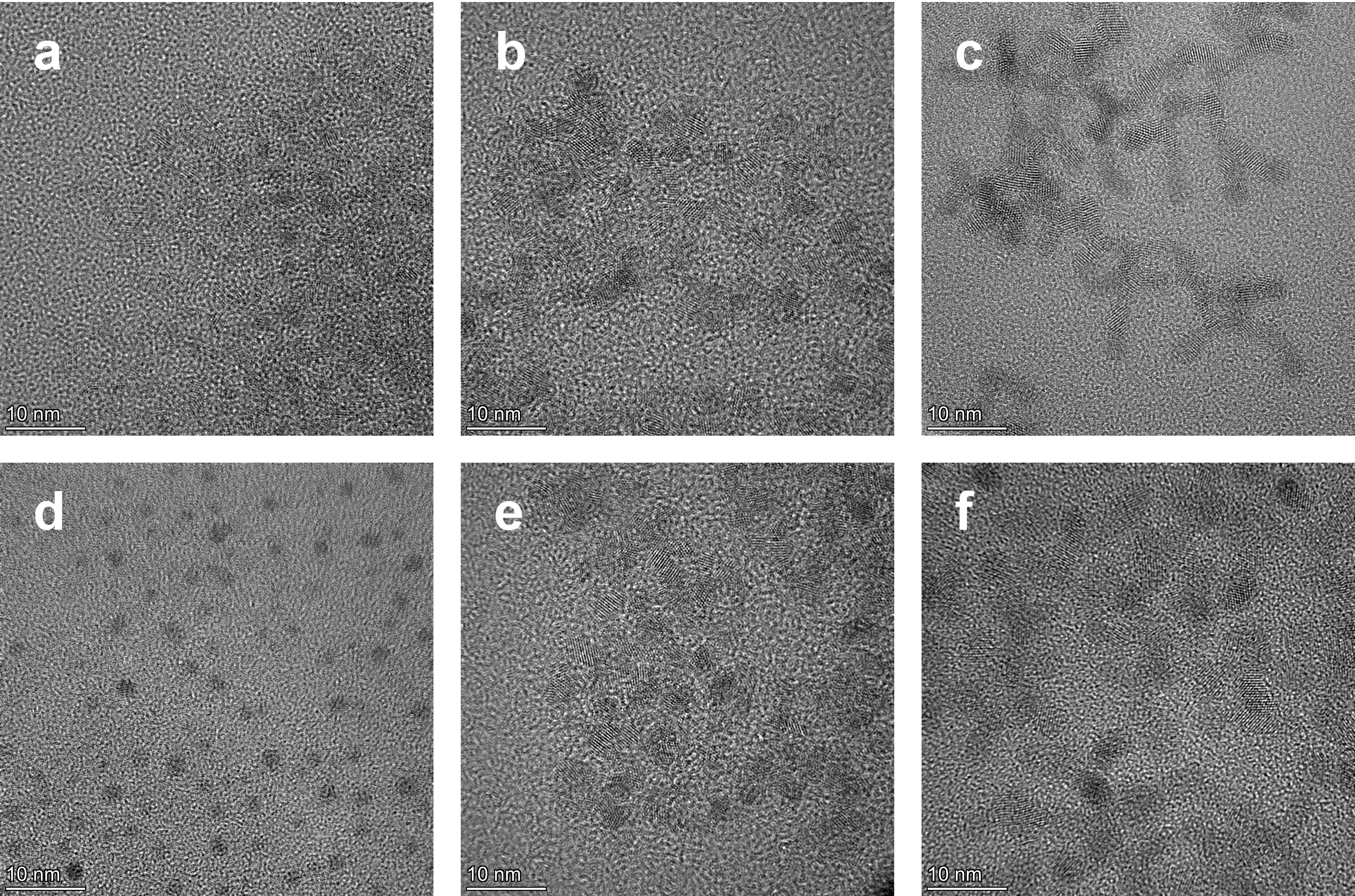


Figure S4-3, HRTEM images of ZnSe QDs synthesized with prepared ZnSe MSC-272 and extra Se. (a) ODESe, 180 °C; (b) ODESe, 240 °C; (c) ODESe+ 2 mmol OA, 240 °C; (d) TOPSe, 180 °C; (e) TOPSe, 240°C; (f) TOPSe+ 2 mmol OA, 240 °C.





Figure S4-4, XRD patterns of ZnSe QDs synthesized with prepared ZnSe MSC-272 and extra TOPSe.


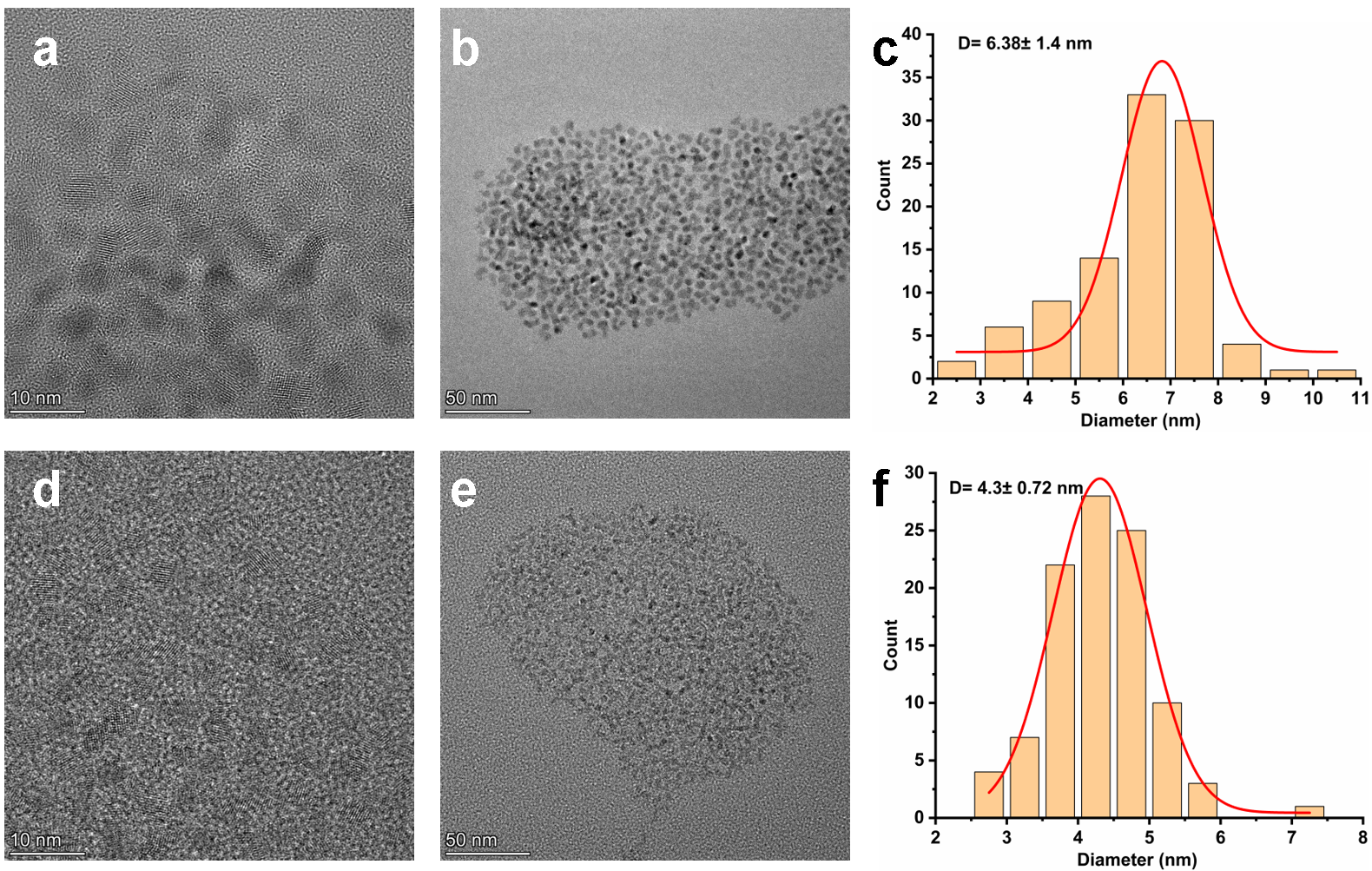


Figure S4-5, TEM images and size distribution statistics of ZnSe QDs synthesized with prepared (a–c) ZnSe MSC-272 and (d–f) molecular precursors at 300 °C, 20 min.


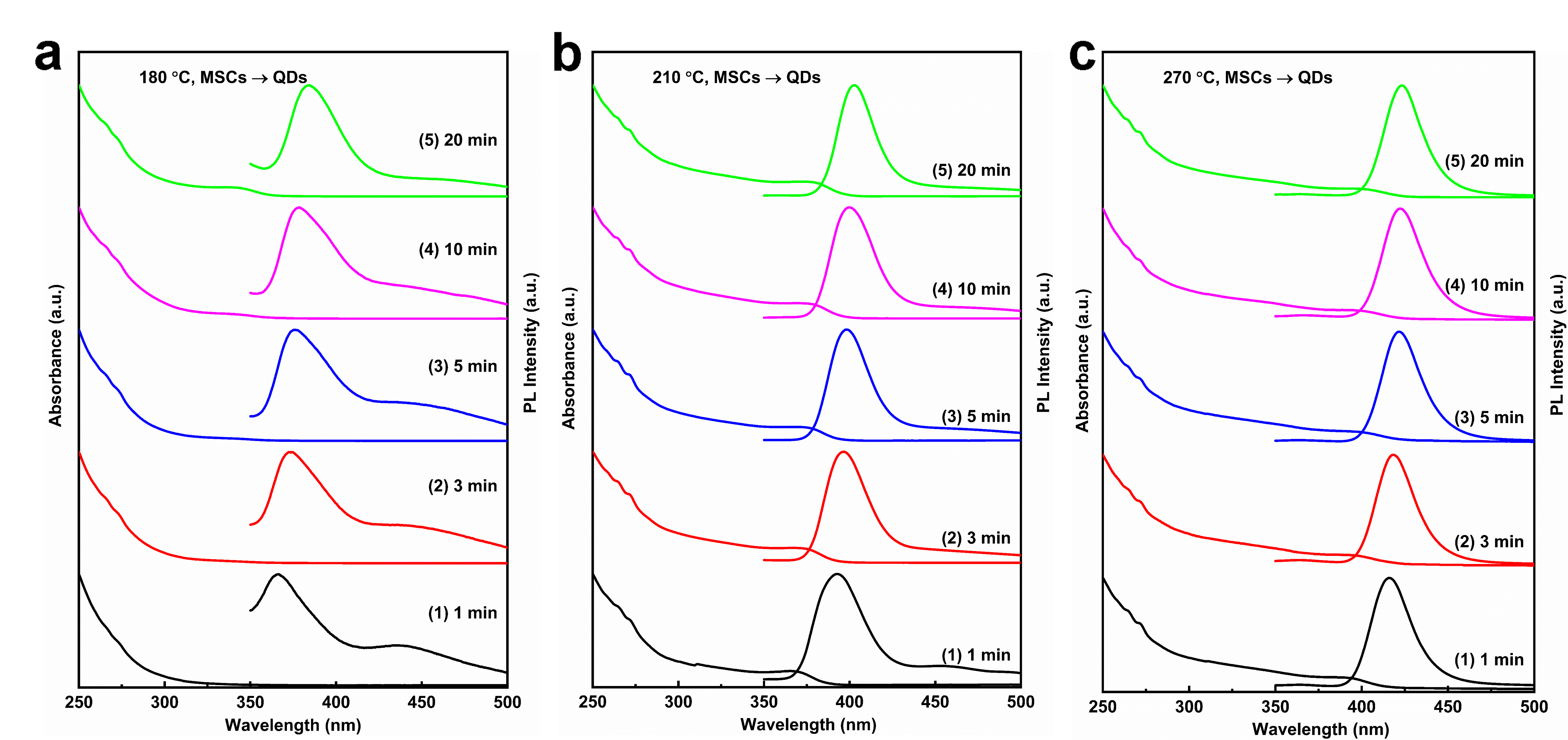


Figure S4-6, Evolution of the UV-vis absorption and PL spectra of ZnSe QDs fabricated from MSCs at (a) 240 °C and (b) 300 °C.





Figure S4-7, PL decay curves of QDs synthesized with different precursors.





Figure S4-8, XRD patterns of ZnSe QDs synthesized with prepared molecular precursors.
